# Sulforaphane as a Photoprotective Agent Against UV-Induced Skin Damage and Carcinogenesis: A Scoping Review

**DOI:** 10.3390/jpm16060319

**Published:** 2026-06-14

**Authors:** Marco Di Filippo, Giovanni Paolino, Matteo Riccardo Di Nicola, Norbert Kiss, András Bánvölgyi, Giulio Bortone, Steven Paul Nisticò, Elia Zampini, Giovanni Pellacani, Carmen Cantisani

**Affiliations:** 1 UOC of Dermatology, Department of Clinical Internal and Cardiovascular Sciences, “Sapienza” University of Rome, 00161 Rome, Italy; marco.difilippo@uniroma1.it (M.D.F.); giuliobortone93@gmail.com (G.B.); steven.nistico@uniroma1.it (S.P.N.); elia.zampini@uniroma1.it (E.Z.); giovanni.pellacani@uniroma1.it (G.P.); 2 Unit of Dermatology, IRCCS San Raffaele Hospital, 20132 Milan, Italy; paolino.giovanni@hsr.it; 3 Istituto Zooprofilattico Sperimentale del Piemonte, Liguria e Valle d’Aosta, 10154 Turin, Italy; matteoriccardo.dinicola@izsplv.it; 4 Department of Dermatology, Venereology and Dermatooncology, Semmelweis University, 1085 Budapest, Hungary; norbert.f.kiss@gmail.com (N.K.); banvolgyi.andras@semmelweis.hu (A.B.)

**Keywords:** sulforaphane, ultraviolet, photoprotection, photocarcinogenesis, Nrf2, oxidative stress, inflammation, broccoli, phytochemical

## Abstract

**Background/Objectives**: Ultraviolet (UV) radiation is a major environmental carcinogen responsible for skin damage through oxidative stress, DNA damage, and inflammation. The nuclear factor erythroid 2-related factor 2 (Nrf2) pathway plays a central role in regulating cellular antioxidant defences against UV-induced damage. This scoping review aims to evaluate the potential role of sulforaphane (SFN), a known Nrf2 inducer, in protecting against UV-induced skin damage and photocarcinogenesis. **Methods**: A literature search was conducted in PubMed and Scopus from inception to 27 January 2026, to identify original experimental studies investigating SFN, glucoraphanin, or broccoli sprout extracts in the context of UV-induced skin damage. Eligible studies included in vitro, ex vivo, in vivo, and human models assessing outcomes related to oxidative stress, inflammation, molecular signalling pathways, and tumour development. Following screening and eligibility assessment, twelve studies were included in the qualitative synthesis. **Results**: The included studies suggest that SFN exerts photoprotective effects across multiple experimental models. In murine studies, SFN and SFN-rich extracts were associated with a reduction in tumour incidence, multiplicity, and volume following UV exposure. In human studies, topical SFN application reduced UV-induced erythema and induced cytoprotective enzyme expression, although clinical evidence remains limited. Mechanistically, SFN consistently activated the Nrf2 pathway, leading to increased expression of antioxidant and phase II detoxifying enzymes, and was associated with modulation of inflammatory responses and inhibition of MAPK/AP-1 signalling. Emerging evidence also indicates potential effects on UV-induced metabolic and epigenetic alterations. **Conclusions**: Current evidence supports a potential role for sulforaphane in mitigating UV-induced skin damage through activation of endogenous defence pathways. However, the available data are predominantly preclinical, and further well-designed clinical studies are needed to clarify its efficacy and translational relevance in humans.

## 1. Introduction

Ultraviolet (UV) radiation is a well-established environmental carcinogen and represents the major aetiologic agent in the development of skin cancers [1,2]. Indeed, UV radiation can act as an initiator and promoter of carcinogenesis as it causes both direct and indirect DNA damage; the former is mainly due to the formation of pyrimidine dimers and pyrimidine–pyrimidone photoproducts in the genome [3], while the latter is caused by the generation of reactive oxygen species (ROS) and reactive nitrogen species (RNS), along with lipid peroxidation, all of which ultimately cause oxidative stress [4,5]. Additionally, exposure to UV radiation can lead to immunosuppression [3,6] and to the induction of nuclear factor κB (NFκB), thereby triggering the production of the pro-inflammatory cytokines IL-1, IL-6 and TNF-α [7].

The skin is endowed with a complex network of defence mechanisms against UV-induced damage, including both enzymatic and non-enzymatic antioxidant systems, which act in concert to preserve redox homeostasis and maintain skin integrity [8]. Among these, the nuclear factor erythroid 2-related factor 2 (Nrf2) is a redox-sensitive transcription factor that acts as a key regulator of cellular antioxidant defences against environmental stressors, including ultraviolet (UV) radiation [9]. Nrf2 has been shown to exert protective effects on skin cells—such as keratinocytes, fibroblasts, and melanocytes—by mitigating UV-induced oxidative damage and maintaining the physiological cellular functions [10].

Therefore, investigating compounds that modulate Nrf2-mediated antioxidant pathways may provide valuable insights for developing pharmacological strategies aimed at delaying skin photoaging and preventing skin carcinogenesis.

In this regard, multiple in vitro and in vivo studies have shown that sulforaphane (SFN), an isothiocyanate mainly generated from the glucoraphanin precursor found in cruciferous vegetables, can function as an inducer of Nrf2 and confer protective effects against UV-induced skin damage and carcinogenesis. These benefits are mediated through several cellular mechanisms, including the upregulation of phase II cytoprotective enzymes and the suppression of inflammatory responses [5,11].

In addition to Nrf2 activation, sulforaphane has been shown to modulate other signalling pathways, including inhibition of activator protein-1 (AP-1), suppression of mitogen-activated protein kinase (MAPK) signalling, and attenuation of pro-inflammatory mediators [12,13].

Despite growing evidence supporting the photoprotective properties of sulforaphane, the mechanistic basis of its action in the context of UV-induced skin damage remains multifaceted.

To the best of our knowledge, this is the first scoping review specifically addressing sulforaphane in the context of UV-induced skin damage and photocarcinogenesis. By integrating data from in vitro, ex vivo, animal, and human studies, this work aims to elucidate how sulforaphane contributes to cutaneous photoprotection and to assess its potential role as a chemopreventive strategy in the context of UV exposure.

## 2. Materials and Methods

### 2.1. Literature Search Strategy

A comprehensive literature search was conducted in the electronic databases PubMed and Scopus from inception to 27 January 2026. The search strategy was designed to identify studies investigating the role of sulforaphane and related compounds in UVR-induced skin damage or skin carcinogenesis. Search terms included combinations of keywords related to sulforaphane, ultraviolet radiation and skin-related outcomes. Specifically, the following search string was used in PubMed: (“Sulforaphane” OR “Glucoraphanin” OR “Broccoli sprout extract” OR “Broccoli sprouts” OR “Broccoli” OR “Isothiocyanate*”) AND (UV OR UVB OR UVA OR “Ultraviolet radiation”) AND (“Skin” OR “Keratinocyte*” OR “Photodamage” OR “Photoaging” OR “Skin cancer” OR “Photocarcinogenesis”). The same combination of keywords and Boolean operators was adapted for use in Scopus to account for database-specific indexing and search fields. Only articles published in English were considered. The inclusion of the broader search term “isothiocyanate*” was intended to maximize sensitivity during study identification; however, studies focusing on isothiocyanates other than sulforaphane were excluded during full-text assessment.

This scoping review was conducted in accordance with the Preferred Reporting Items for Systematic Reviews and Meta-Analyses extension for Scoping Reviews (PRISMA-ScR) [14]. The completed PRISMA-ScR checklist is provided in the Supplementary Materials (Table S1). No protocol was registered.

### 2.2. Study Selection

**Figure 1:**
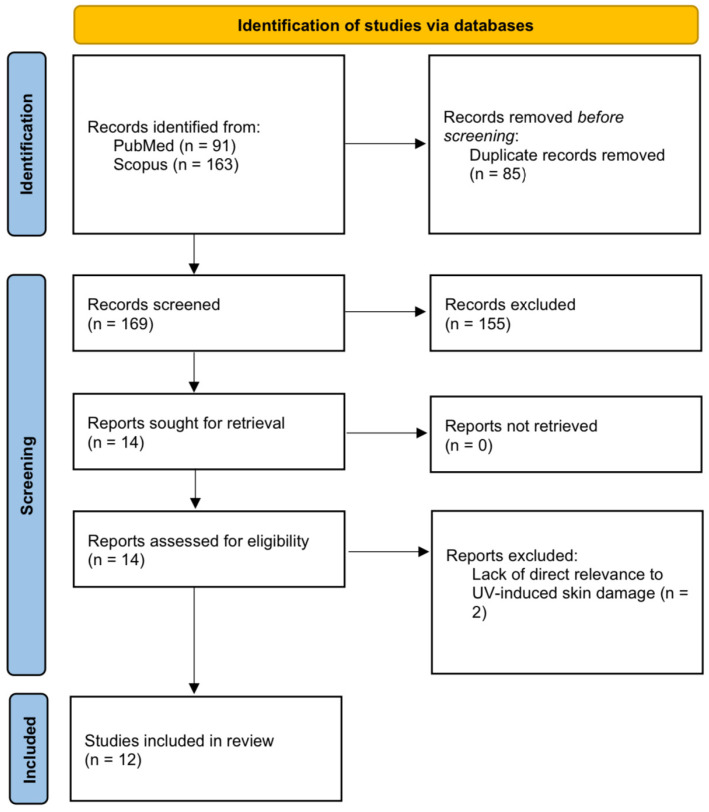
PRISMA flowchart showing identification, screening and inclusion of articles.

The records identified through database searches (PubMed and Scopus) were imported into a management system, and duplicates were removed. The study selection process was performed independently by two reviewers. Titles and abstracts were screened for relevance. Studies that did not meet the inclusion criteria were excluded at this stage. Full-text versions of the remaining articles were retrieved and assessed for eligibility by the same two reviewers. Reasons for exclusion at the full-text stage were documented. Any disagreements were resolved through discussion or consultation with a third reviewer. A PRISMA flowchart summarizes search counts for inclusions and exclusions and reasons for study exclusions (Figure 1).

### 2.3. Eligibility Criteria

Studies were considered eligible for inclusion if they were original experimental investigations analyzing the effects of sulforaphane, its precursor glucoraphanin, or sulforaphane-rich broccoli sprout extract (BSE) in the context of ultraviolet radiation-induced skin damage. The focus on cruciferous vegetables is justified by their distinctive content of glucoraphanin, the direct precursor of sulforaphane, which is largely confined to Brassicaceae species. Among these vegetables, broccoli represents the most extensively investigated and quantitatively relevant source of sulforaphane, as reflected by the large body of preclinical and clinical studies employing broccoli-derived preparations [15]. Eligible studies included in vitro, ex vivo, in vivo, and human experimental models in which ultraviolet radiation (UV, UVB, or UVA) represented a central experimental factor contributing to cutaneous injury, inflammation, photoaging, or photocarcinogenesis. Only studies employing skin-relevant models—such as human keratinocytes, ex vivo human skin, animal models of UV-induced skin damage or photocarcinogenesis, or controlled UV exposure in human skin—were included. Furthermore, studies were required to assess UV-related cutaneous outcomes, including oxidative stress, inflammatory responses, apoptosis, photoaging-associated markers, modulation of molecular signalling pathways (such as Nrf2, AP-1, or MAPK), or the development of UV-induced skin tumours.

Studies were excluded if they were review articles or other non-original publications, or if UV radiation was not a central component of the experimental design. Additionally, articles were deemed not eligible when skin carcinogenesis or tissue damage was induced through non-UV mechanisms, such as chemically induced models, or when skin-specific outcomes were not clearly reported. In addition, studies in which sulforaphane was only marginally evaluated, or where its effects could not be clearly distinguished from those of other kinds of intervention, were not included in our review.

### 2.4. Data Extraction

From each included study, relevant data were extracted, namely the experimental model, type and route of sulforaphane administration, UV exposure parameters, and main biological outcomes related to skin damage or photoprotection. Data extraction was performed to enable qualitative synthesis of the evidence.

## 3. Results

### 3.1. Study Characteristics

A total of 169 records were identified through database searching. After duplicate removal, records were screened by title and abstract. Fourteen articles were retrieved for full-text assessment. Two studies were excluded at the full-text stage because UV exposure was not central to the experimental design. Ultimately, twelve studies fulfilled the inclusion criteria and were included in the qualitative synthesis.

### 3.2. Characteristics of Included Studies

The main characteristics of the included studies are summarized in Table 1. These studies comprise a combination of in vitro, ex vivo, in vivo, and human experimental studies. Experimental models included human keratinocyte cell lines (HaCaT cells), ex vivo human full-thickness skin, UV-exposed hairless mouse models (predominantly SKH1), and controlled UV exposure in healthy human volunteers.

Ultraviolet radiation was used as a primary experimental insult in all included studies, with UVB being the most frequently employed wavelength.

Sulforaphane was administered either as a purified compound or in the form of glucoraphanin or sulforaphane-rich broccoli sprout extracts, delivered through topical or oral routes depending on the study design.

### 3.3. Prevention and Inhibition of Skin Photocarcinogenesis

SFN has been shown to significantly impede the development of skin tumours following UV insult. For example, in high-risk SKH1 hairless mouse models, topical application of BSE, containing SFN, reduced tumour incidence, multiplicity and total tumour volume by approximately 50% [16]. This protection seems consistent regardless of the delivery method: in this regard, dietary administration of glucoraphanin (the natural precursor of SFN) resulted in a 25% reduction in tumour incidence and a 70% reduction in tumour volume in SKH1 hairless mice with prior chronic UV irradiation. Notably, the most significant effects were observed on larger tumours [17].

Furthermore, SFN was found to be effective across all stages of the complex process of photocarcinogenesis, namely initiation, promotion, and progression, with the most striking effects yielded during the earlier stages of tumour development [18].

The chemopreventive effect of SFN is consistently highlighted throughout the articles we retrieved.

In this regard, topical treatment of mouse skin with SFN has been shown to lead to a marked reduction in the multiplicity and total burden of UVB-induced squamous cell carcinomas (SCCs), suggesting its potential as a long-term preventive strategy [12].

### 3.4. Acute Photoprotection and Erythema

In a study carried out by Talalay et al., graded UV dosages were delivered to healthy human volunteers and the consequent erythema was measured; when topical treatment with SF-containing BSE was applied, a reduction in UV-induced erythema by an average of 37.7% was highlighted, with some individuals experiencing a reduction as high as 78% [5]. Of note, this protection is not a “sunscreen effect” because SFN is almost transparent at 311 nm, which is the wavelength at which the UV radiation delivered in this study was centred [5]. This protection is uniquely durable, persisting for up to 72 h, probably due to the synthesis of long-lived proteins [5].

Recent evidence also suggests that oral supplementation with glucoraphanin can modulate the cutaneous environment, increasing the expression of cytoprotective genes directly in human skin in vivo. However, while this intervention has shown an alteration of cutaneous biochemical markers, it did not lead to a statistically significant reduction in clinical erythema in this specific short-term pilot study [19].

### 3.5. Anti-Inflammatory Mechanisms

In both human keratinocytes (HaCaT cells) and animal models, SFN treatment has been found to suppress the production of pro-inflammatory cytokines such as IL-1 and IL-6 following UV radiation [11,20]. In this regard, oral administration of SFN in mice was shown to attenuate UVB-induced skin thickening and hyperplasia, primarily by inhibiting the expression of cyclooxygenase-2 (COX-2), thereby reducing the release of prostaglandin E2 (PGE2) [20]. This evidence is supported by other studies, like the one carried out by Saw et al. [11], in which the authors observed that SFN effectively normalized the thickness of the skin in Nrf2-knockout mice, after UVB exposure. In order to explain this phenomenon, the authors hypothesized the presence of secondary non-Nrf2-mediated anti-inflammatory pathways.

However, the role of Nrf2 is crucial in order for SFN to display its chemopreventive properties, as it was highlighted by the aforementioned study involving Nrf2-knockout mice. These mice, which lack the Nrf2 protein, were significantly more susceptible to UVB-induced inflammation and cell death and failed to receive the photoprotective benefits of SFN that were clearly seen in wild-type mice [11].

### 3.6. The Nrf2 Pathway and Phase II Enzymes

The primary mechanism behind SFN’s efficacy is its role as a potent Nrf2 inducer, which leads to the activation of antioxidant and phase II detoxifying enzymes [9,21,22]. Of note, the exposure of human and murine keratinocytes to SFN has been linked to a rise in intracellular levels of glutathione and Nrf2 target genes, such as NQO1, which codes for a marker enzyme of the phase II response [16,23].

Furthermore, in ex vivo human full skin, SFN was found to prevent both UVR-induced depletion of catalase, which is a crucial antioxidant enzyme, and apoptosis, by inhibiting caspase-3 activation [23].

### 3.7. Inhibition of AP-1 and MAPK Signalling

Beyond its antioxidant induction, SFN provides a direct molecular defence by inhibiting Activator Protein-1 (AP-1), a transcription factor known to be a key mediator of UV-induced non-melanoma skin cancer (NMSC) [12,21]. SFN directly binds to specific cysteine residues in the DNA-binding domains of c-Fos and c-Jun (members of the AP-1 family), physically preventing them from attaching to DNA [12,21].

Additionally, in a study carried out by Chaiprasongsuk et al. [9], SFN—as a potent Nrf2 activator—attenuated UVA-induced MAPK activation in both keratinocyte cultures and mouse skin; in HaCaT cells, SFN pre-treatment reduced the phosphorylation of ERK, JNK and p38 at 15 min following the final UVA exposure and reduced phosphorylation of c-Fos and c-Jun. Consistently, topical application of SFN to BALB/c mouse dorsal skin, before repeated UVA irradiation, markedly lowered the levels of phosphorylated ERK, JNK and p38. Of note, the inhibition of MAPK phosphorylation correlated with a significant reduction in matrix metalloproteinase-1 (MMP-1) expression and with the preservation of collagen I in the dermis. This finding confirms that SFN mitigates UVA mediated extracellular matrix degradation through MAPK/AP-1 pathway suppression [9].

### 3.8. Metabolic and Epigenetic Rewiring

By activating the Nrf2 signalling axis, SFN drives a robust NRF2-mediated oxidative stress response that upregulates canonical detoxifying genes and suppresses pro inflammatory NF-κB pathways. This Nrf2 activation has been linked to epigenetic remodelling. Integrated multiomics analysis—both in vitro and in SKH1 hairless mice—demonstrated that SFN could at least partially reverse UVB-induced metabolic, transcriptional and epigenetic alterations, supporting a coordinated Nrf2-dependent metabolic epigenetic rewiring that underlies its chemopreventive efficacy [18,22].

**Table 1:** Characteristics of studies investigating sulforaphane in UV-induced skin damage and carcinogenesis. *Abbreviations* BSE, Broccoli sprout extract; CAT, Catalase; γGCS, γ-glutamylcysteine-synthetase; GR, glucoraphanin; GSH, glutathione; HO-1, heme oxygenase 1; HPD, hispidulin; iNOS, inducible nitric oxide synthase; IL-1β, interleukin-1β; KO, knock-out; MAPK/AP-1, mitogen- activated protein kinase/activator protein 1; MED, minimum erythematous dose; MMP-1, Matrix metalloproteinase-1; MPO, myeloperoxidase NQO1, NAD(P)H quinone oxidoreductase 1; Nrf2, nuclear factor erythroid-2 related factor 2; PEITC, phenylethyl isothiocyanate; SFN, sulforaphane; tBHQ, tert-butylhydroquinone; TNFα, tumour necrosis factor α; TRE, TPA (12-O-tetradecanoylphorbol 13-acetate)-response element); WT, wild-type; 8-OHdG, 8-hydroxy-29-deoxyguanosine. * Reported doses refer to glucoraphanin precursor content as described in the original studies and should not be interpreted as directly equivalent to bioavailable SFN, since conversion efficiency depends on myrosinase activity, formulation characteristics, processing conditions, route of administration, and gastrointestinal metabolism.

**Sulforaphane, Topical Administration**							
**Author (Year)**	**Study model**	**UV exposure**	**Intervention**	**Dose**	**Route**	**Main outcomes**	**Key findings**
Kleszczyński et al. (2013) [23]	In vitro HaCaT keratinocytes + ex vivo full-thickness human skin	UVB and UVA and UVC (300 mJ/cm^2^)	SFN and PEITC	Culture medium containing SFN or PEITC at final concentrations of 5, 10 or 25 μM	Topical	Induction of antioxidative and Nrf2 target genes. Reduction in UVR-induced structural damage in the epidermis.	SFN led to induction of antioxidative (CAT) and Nrf2 target genes (γGCS, HO-1 and NQO1), reduced UVR-induced structural damage in the epidermis, prevented UVR-induced depletion of CAT and inhibited apoptotic caspase-3 activation in human full skin.
Dickinson et al. (2009) [12]	SKH-1 hairless mice	UVB 3 times a week for 25 weeks, initiated at 0.54 kJ/m^2^ and increased each week until 1.65 kJ/m^2^ at week 5 and maintained for the rest of the experiment	SFN	0.3 μmol per ear; 1 or 2.5 μmol per back	Topical	Skin carcinogenesis, AP-1 luciferase inhibition, c-Fos binding inhibition.	Topical SFN markedly reduced tumour multiplicity and tumour burden. SFN inhibits UVB-induced AP-1 luciferase in vivo. SFN inhibits nuclear binding of c-Fos to the MMP-1 TRE after UVB exposure.
Chaiprasongsuk et al. (2017) [9]	HaCaT keratinocytes + BALB/c mice	UVA (4 J/cm^2^) for HaCaT cells UVA at 10 J/cm^2^/session three times per week for 2 weeks (a total dose of 60 J/cm^2^) for BALB/c mice	SFN, HPD	0.6 mM/cm^2^ (SFN)	Topical	MMP-1 modulation, Nrf2 activation, protection against UVA-induced connective tissue damage.	Depletion of Nrf2 augmented UVA-induced MMP-1 via modulation of MAPK/AP-1 Signalling in HaCaT keratinocytes. SFN treatment dramatically induced the nuclear Nrf2 levels and its target antioxidant proteins in mouse epidermis, with decrease in 8-OHdG formation after irradiation. With topical application of HPD or SFN 1 h prior to each UVA irradiation, there was a pronounced reduction in MMP-1 expression, an increase in collagen levels, and a marked reduction in epidermal thickness. Nrf2 activators promote reduction in MMP-1 Activity via MAPK/AP-1 signalling cascade.
Saw et al. (2011) [11]	Nrf2 KO and WT C57BL/6 mice	Single dose of UVB (300 mJ/cm^2^)	SFN	100 nmol in 100 μL acetone	Topical	Inflammation, apoptosis.	SFN-mediated photoprotection required functional Nrf2: Nrf2 (-/-) mice were more susceptible to UVB-induced skin inflammation and thickening. The number of sunburn cells per field in the KO mice was greater than that in the WT. However, SFN treatment was also found to be effective in normalizing the thickness of the skin back to its basal level in KO mice too, probably via other non-Nrf2 pathways such as direct anti-inflammatory pathways.
Li et al. (2020) [18]	SKH-1 hairless mice	UVB radiation of 60 mJ/cm^2^ two times per week.	SFN	2 μmol SFN in 200 μL acetone	Topical	Tumour incidence, tumour number, Epigenetic and transcriptomic changes.	The SFN group had significantly fewer tumours with decreased total tumour volume and tumour number (*p*-value < 0.05). SFN led to the upregulation of genes downregulated by UVB and downregulation of genes upregulated by UVB. The same applied for DNA methylome alterations.
**Sulforaphane, Oral Administration and In Vitro Studies**							
**Author (Year)**	**Study model**	**UV exposure**	**Intervention**	**Dose**	**Route**	**Main outcomes**	**Key findings**
Shibata et al. (2010) [20]	HaCaT keratinocytes + HR1 hairless mice	UVB (50 mJ/cm2) (for HaCaT cells) UVB (200 mJ/cm^2^) on days 9, 11 and 13 (for the animal study)	SFN	0–25 μM (in vitro), 1–2.5 mg/day for 14 days (mice)	In vitro and Oral	Cutaneous anti-inflammatory mechanism of SFN, in vivo inhibition of skin inflammation.	Both the UVB-induced skin thickness and the COX-2 protein expression were suppressed by oral administration of SFN to mice.
Zhu et al. (2004) [21]	HCL14 cells	UVB (peak emission of 313 nm)	SFN (and tBHQ)	0–10 μM	In vitro	Inhibition of AP-1 activation, enzyme induction.	SFN (and tBHQ) significantly elevated phase II enzyme activity and GSH levels in human HCL14 keratinocytes. SFN (but not tBHQ) inhibited the UVB-induced AP-1 activation by inhibiting AP-1 binding activity to its target DNA.
Li et al. (2022) [22]	HaCaT keratinocytes	UVB (0, 1, 5, 10, and 15 mJ/cm^2^) every 7 days for 10 cycles	SFN	10 μM	In vitro	SFN-induced metabolic, transcriptomic and DNA methylation changes.	SFN attenuated UVB-induced metabolic, genetic and epigenetic dysregulation.
Broccoli Sprout Extract *, Topical Administration							
**Author (Year)**	**Study model**	**UV exposure**	**Intervention**	**Dose**	**Route**	**Main outcomes**	**Key findings**
Talalay et al. (2007) [5]	Human healthy volunteers + Mice	Narrow-band UV (centred at 311 nm)	BSE	100, 200, 400, or 600 nmol SF as BSE in 25 mL of 80% acetone/20% water on 3 days at 24 h intervals (human healthy volunteers) Three doses of BSE containing 0.5 μmol of SF in 50 mL of 80% acetone/20% water (vol/vol) applied to the caudal area (mice)	Topical	UV-induced erythema, inflammation, edema.	SFN-rich BSE led to the induction of NQO1 and the inhibition of the UVR-dependent MPO activity. UV-induced damaging effects (increased thickness, edema and inflammation in mice) were averted by prior treatment of mouse skin with BSE. Significant protection against UV erythema in human volunteers.
Dinkova-Kostova et al. (2006) [16]	SKH-1 hairless mice + HaCaT keratinocytes	Mixture of UVB and UVA (30 mJ/cm^2^/session twice a week for 20 weeks).	BSE	0.3 or 1 μmol in 100 μL acetone/water (80%/20%) (mice), 0–10 μM (HaCaT keratinocytes)	Topical	Tumour burden, incidence, and multiplicity.	SFN-containing extracts reduced UVB-induced tumour burden, incidence and multiplicity and delayed tumour appearance. Exposure to SFN elevates NQO1 and GSH and protects against UV-radiation- generated oxidative stress in keratinocytes. Topical application of BSE as a source of SFN elevates NQO1 in mouse skin. SFN inhibits iNOS upregulation.
Broccoli Sprout Extract *, Oral Administration							
**Author (Year)**	**Study model**	**UV exposure**	**Intervention**	**Dose**	**Route**	**Main outcomes**	**Key findings**
Dinkova-Kostova et al. (2010) [17]	SKH-1 hairless mice	UV radiation (30 mJ cm^2^ of UVB) twice a week for 17 weeks	BSE	10 μmol of GR per 3 g of diet	Oral	Tumour incidence, multiplicity and volume.	Dietary SFN reduced tumour incidence, multiplicity, and volume compared to the controls.
Chien et al. (2025) [19]	Healthy human volunteers	2 × MED of UVB	Glucoraphanin, curcumin or both	Crucera-SGS^®^ with TrueBroc^®^ broccoli seed extract (Brassica Protection Products, LLC, Baltimore, MD, USA); 9 capsules (450 mg or 1.03 mmol GR) per day for 10 days	Oral	UV-induced erythema, Biomarker modulation.	GR was associated with a significant increase in mRNA copy number for NQO1 in the skin and expression of HO-1, IL-1β, and TNF-α were reduced.

## 4. Discussion

Our review synthesizes the available evidence on the photoprotective effects of sulforaphane against UV-induced skin damage and photocarcinogenesis. Overall, the included studies suggest that sulforaphane may exert protective effects across multiple experimental settings, including in vitro keratinocyte models, ex vivo human skin, animal models, and a limited number of human studies. Collectively, these data support a biologically plausible role for sulforaphane in modulating oxidative stress, inflammatory responses, and molecular pathways involved in UV-mediated cutaneous injury.

A central finding emerging from the available literature is the key role of Nrf2 signalling in mediating the effects of sulforaphane. Several studies showed that sulforaphane activates Nrf2 and enhances the expression of downstream cytoprotective and antioxidant enzymes, including NQO1, HO-1, catalase, and enzymes involved in glutathione homeostasis. Through this mechanism, sulforaphane appears to strengthen the endogenous cellular defence system against UV-induced oxidative stress. Importantly, evidence from Nrf2 knockout models indicates that an intact Nrf2 pathway is required for the full photoprotective and anti-inflammatory activity of sulforaphane in vivo, thereby supporting the mechanistic relevance of this pathway [11]. The role of SFN as a Nrf2 inducer is linked to its ability to modify Keap1 cysteine residues, which results in a conformational change in the associated motif of Keap1–Nrf2 that allows the dissociation of Nrf2 from Keap1 and the subsequent nuclear translocation of Nrf2 [24].

Beyond redox regulation, sulforaphane also appears to modulate pathways involved in inflammation, extracellular matrix remodelling, and tumour promotion. In particular, the available evidence indicates suppression of MAPK/AP-1 signalling after UV exposure, with downstream effects on MMP-1 expression, collagen preservation, and tissue injury. Furthermore, the capacity of SFN to potentially preserve collagen and prevent skin wrinkling by inhibiting MMP-1 and MMP-3 expression could also prove to be beneficial for anti-ageing purposes. However, it is crucial to consider the hormetic nature of SFN, as its protective effects against UV-induced damage are primarily observed at low concentrations, whereas higher doses may lead to increased cytotoxicity [25].

Anti-inflammatory activity has also been observed, including inhibition of COX-2 and attenuation of UV-induced cytokine responses [9,12,20].

The potential relevance of these effects to photocarcinogenesis is particularly noteworthy. In murine models, topical sulforaphane or sulforaphane-rich broccoli sprout preparations, as well as dietary glucoraphanin-rich interventions, were associated with reductions in tumour incidence, multiplicity, and tumour burden after chronic UV exposure. These findings suggest that sulforaphane may interfere with multiple phases of UV-driven skin carcinogenesis, although this interpretation remains largely preclinical [12,16,17,18]. The reduction in tumour incidence and burden observed in these murine models is further supported by a recent systematic review [26], which highlights SFN as a significant chemopreventive candidate due to its consistent ability to impair tumour progression across various experimental designs.

Human evidence remains limited but suggestive. Topical sulforaphane-rich preparations reduced UV-induced erythema and enhanced cytoprotective responses in human skin. In contrast, oral glucoraphanin supplementation was associated with biomarker modulation without a statistically significant reduction in clinical erythema. Taken together, these findings indicate measurable molecular effects in human skin, while highlighting that short-term molecular changes do not necessarily translate into clinically meaningful outcomes [5,19].

Another relevant aspect is the possibility that sulforaphane may influence broader molecular alterations induced by chronic UV exposure. Recent metabolomic, transcriptomic, and DNA methylation data suggest that sulforaphane may partially counteract UV-induced molecular dysregulation in keratinocytes and in animal models. Although these findings further support its chemopreventive potential, their clinical significance remains uncertain and should not be overinterpreted in the absence of long-term human data [18,22].

From a conceptual perspective, these observations may also intersect with the broader framework of field cancerisation in chronically sun-exposed skin. By enhancing endogenous defence mechanisms and attenuating pro-carcinogenic signalling, sulforaphane could theoretically help limit the accumulation of subclinical molecular alterations within UV-damaged epidermal fields. However, this remains a hypothesis rather than a demonstrated effect, since none of the included studies directly assessed field cancerisation as a clinical or histopathological endpoint [27,28].

A critical translational issue in the development of sulforaphane-based photoprotection strategies is its intrinsic chemical instability: sulforaphane is highly reactive and susceptible to degradation under conditions of heat, oxidation, and prolonged aqueous exposure, which may substantially affect its bioavailability and biological activity [15]. This property complicates both experimental reproducibility and clinical dose standardization across studies.

An additional layer of complexity derives from the distinction between direct sulforaphane administration and glucoraphanin-containing preparations, such as BSE. In plant tissues, glucoraphanin is stored as a relatively stable precursor and is converted into bioactive sulforaphane through hydrolysis by the enzyme myrosinase [15,16,17]. The efficiency of this conversion is highly variable and depends on processing methods, preservation of enzymatic activity, formulation characteristics, and route of administration [15,17,19]. This distinction is particularly relevant when comparing topical with oral interventions. Oral administration may permit glucoraphanin conversion during mastication and gastrointestinal digestion, potentially aided by gut microbiota, although interindividual variability remains substantial. By contrast, topical delivery of glucoraphanin-based formulations may provide less predictable conversion unless active myrosinase is preserved within the preparation or sulforaphane itself is directly administered in a stabilized formulation [15].

These considerations indicate that the central practical challenge lies not simply in establishing whether sulforaphane exerts photoprotective effects but in determining how it can be delivered in a stable, bioavailable, and reproducible manner. Future translational studies should therefore prioritize the development of optimized formulation strategies, rigorous assessment of conversion efficiency, and direct comparative evaluation of active sulforaphane versus precursor-based preparations.

This review has several limitations. The number of eligible studies was small, and substantial heterogeneity was observed across UV sources, dosing regimens, formulations, routes of administration, experimental models, and outcome measures. Moreover, the evidence base is still heavily weighted towards preclinical studies, whereas human data remain sparse and largely restricted to short-term surrogate endpoints. These factors, together with substantial heterogeneity in compound stability, precursor-to-active conversion efficiency, and formulation-dependent bioavailability, limit direct comparability across studies and preclude firm conclusions regarding the optimal formulation, dose, route, and treatment schedule for clinical use.

Overall, the available evidence supports sulforaphane as a promising candidate for UV-related skin protection, with effects spanning antioxidant defence, inflammatory signalling, extracellular matrix preservation, and tumour-related pathways. Nevertheless, the current level of evidence supports mechanistic plausibility and preclinical efficacy more strongly than established clinical effectiveness. Further well-designed human studies are needed to clarify its translational relevance and to determine whether these molecular and experimental effects can meaningfully reduce photodamage and UV-related skin carcinogenesis.

## Data Availability

No new data were created or analyzed in this study. Data sharing is not applicable to this article.

## Supplementary Materials

The following supporting information can be downloaded at: https://www.mdpi.com/article/10.3390/jpm16060319/s1, Table S1: PRISMA-ScR Checklist [29].
